# Cholesterol Degradation and Production of Extracellular Cholesterol Oxidase from* Bacillus pumilus* W1 and* Serratia marcescens* W8

**DOI:** 10.1155/2019/1359528

**Published:** 2019-04-28

**Authors:** Hasina Wali, Fazal Ur Rehman, Aiman Umar, Safia Ahmed

**Affiliations:** ^1^Department of Microbiology, Quaid-i-Azam University, Islamabad 45320, Pakistan; ^2^Department of Microbiology, University of Balochistan, Quetta 87300, Pakistan

## Abstract

Cholesterol is a waxy substance present in all types of the body cells. The presence of higher concentration of low density lipoprotein (LDL) is characterized by abnormal cholesterol level and is associated with cardiovascular diseases which lead to the development of atheroma in arteries known as atherosclerosis. The transformation of cholesterol by bacterial cholesterol oxidase can provide a key solution for the treatment of diseases related to cholesterol and its oxidized derivatives. Previously isolated bacteria from oil-contaminated soil were screened for cholesterol degradation. Among fourteen, five isolates were able to utilize cholesterol. Two strains* Serratia marcescens* W1 and* Bacillus pumilus* W8 using cholesterol as only carbon and energy source were selected for degradation studies. Several parameters (incubation time, substrate concentration, pH, temperature, and different metal ions) for cholesterol decomposition by the selected bacterial strains were evaluated. Maximum cholesterol reduction was achieved on the 5^th^ day of incubation, 1g/L of substrate concentration, pH 7, in the presence of Mg^2+^ and Ca^2+^ ions, and at 35°C. Cholesterol degradation was analyzed by enzymatic colorimetric method, thin layer chromatography (TLC), and high-performance liquid chromatography (HPLC). Under optimized conditions 50% and 84% cholesterol reduction were recorded with* Serratia marcescens* W1 and* Bacillus pumilus* W8, respectively. Cholesterol oxidase activity was assayed qualitatively and quantitatively. The results revealed that* Serratia marcescens* W1 and* Bacillus pumilus* W8 have great potential for cholesterol degradation and would be regarded as a source for cholesterol oxidase (CHO).

## 1. Introduction

Cholesterol is a steroid generally found in human and animal tissues and also in plasma lipoprotein either as free cholesterol or linked with fatty acids. It plays major role in vitamin D and hormones production to build healthy cells and also substances to aid digestion [1]. The level of cholesterol in the body is regulated and maintained by cholesterol synthesis and absorption, which have a reciprocal relationship, and by elimination of cholesterol into the bile. That is why, the proper evaluation of synthesis, absorption, and trafficking of cholesterol all over the body is critical to health research [2]. Several diseases emerge due to imbalance in these processes. In pathological process, cholesterol plays a major role in the development of atherosclerosis of main arteries causing cerebrovascular, peripheral, and coronary vascular diseases [1]. Furthermore, cholesterol rich diet has strong association with prevalence of coronary heart diseases (CHD). The epidemiological studies also demonstrated a strong link between the risk of coronary heart disease and plasma cholesterol concentration [3].

The oxidation products of cholesterol are known as oxysterols, which are considered to be involved in the pathogenesis of atherosclerotic lesions; the major oxidation product of cholesterol is 7-ketocholesterol which is more atherogenic than cholesterol [4]. Both* in vitro *[5] and* in vivo *[6, 7] inflammatory effects have been shown by this oxysterol. Its cytotoxic and inflammatory effects on the cells are proven and also reported in the pathogenesis of several neurodegenerative diseases [7, 8], heart diseases [6, 9], cancer [10], and Parkinson's disease [8] as well as age-related macular degeneration [8, 11].

In developed countries atherosclerosis is the leading cause of death. It develops slowly and usually asymptomatically until brittle arterial plaques rupture and causes a stroke or heart attack [12]. In the artery walls, high concentrations of oxidized lipoprotein are associated with development of atherosclerosis [13]. Macrophages and endothelial cells make efforts to remove these accumulations, but are incapable of carrying out this function. As a result, they transform into defective foam cells due to saturation with fatty material. More immune cells are attracted, due to inflammation and fibrosis which further add to instead of resolving the storage problem [14].

Many bacterial species have been reported to be involved in biodegradation of cholesterol by means of bifunctional, flavin adenine dinucleotide containing cholesterol oxidase which oxidizes the cholesterol and produces 4-cholesten-3-one, with reduction of oxygen to hydrogen peroxide [15, 16]. The degradation of cholesterol by* Mycobacterium, Rhodococcus*,* Brevibacterium*,* Streptomyces*, and some other Gram positive as well as Gram negative genera including* Comamonas*,* Burkholderia, Pseudomonas*, and* Chromobacterium *has been reported [17–22].

Significant attention has been received by cholesterol oxidase due its wider use for detection of cholesterol in food and blood samples, which has direct implication in lipid disorders including coronary heart diseases and atherosclerosis. Additionally, cholesterol oxidase is used in production of steroids [23]. Although in human body cholesterol is a vital substance, with age due to catabolic insufficiency these compounds start to accumulate, which is associated with certain age-related diseases. As the endogenous system fails to remove these compounds, the enzyme replacement therapy is an alternative therapeutic option in which microbial enzymes are used to degrade the pathogenic compounds [24, 25].

The present study was aimed at screening the potent cholesterol decomposing bacteria, isolated from oil-contaminated soil, and evaluating their potential for cholesterol oxidase production.

## 2. Material and Methods

### 2.1. Chemicals

The substrate cholesterol was purchased from VWR Life Science AMRESCO® (Ultra-Pure Grade). In all experiments M9 salt medium was used as growth medium. The composition of growth medium was 15 g KH_2_PO_4_, 64 g Na_2_HPO_4_.7H_2_O, 5.0 g NH_4_Cl, 2.5 g NaCl, 2 mL of 1M MgSO_4_, and 0.1 mL of 1M CaCl_2_ in 1000 mL of distilled water.

### 2.2. Screening of Cholesterol Degrading Bacteria

Several bacterial isolates from oil-contaminated soil were screened on M9 salt agar plates containing 0.1% cholesterol as the only carbon source. Cholesterol plates were streaked with cultures and incubated in an incubator at 37°C for 7 days. The potentiality of bacteria to utilize cholesterol was evaluated via the growth of bacteria on these plates.

### 2.3. Optimization Studies

To optimize several degradation parameters, throughout the study shake flask fermentation experiments were carried out with 100 mL of fermentation broth (M9 medium) in conical flasks (250 mL) containing 0.1% cholesterol and inoculated with 2% (v/v) bacterial culture and incubated at 30°C. Different factors affecting the degradation of cholesterol were studied, such as incubation time (1 to 6 days), incubation temperature (25 to 45°C), pH values (pH 3 to 9), different metal ions (MgSO_4_, CaCl_2_, BaCl_2_, CoCl_2_), and different substrate concentrations (0.5 to 2 g/L).

### 2.4. Analysis of Cholesterol Degradation

Enzymatic colorimetric cholesterol oxidase peroxidase method was used to calculate the cholesterol degradation [26]. Growth was monitored at 600 nm using spectrophotometer. Cholesterol estimation kit (Merck) was used in order to perform cholesterol assay. All the reagents were mixed well according to manufacturer's instructions. In the reaction mixture 10 *μ*L of cell free supernatant (CFS) was added, mixed by inversion, and then incubated at 37°C for 10 minutes. The absorbance of test and standard was checked against the blank at* ƛ* 505 nm, and concentration of cholesterol was determined by the following formula.

(1) Cholesterol  mg/dL=Absorbance  of  testAbsorbance  of  standard×Conc.  of  standard  mg/dL

Further the percent cholesterol degradation was calculated from the cholesterol concentrations at the start of the experiment and after certain time intervals. The culture supernatant of the cholesterol degradation experiments was extracted with a mixture of hexane/isopropanol (3:2), dried and redissolved in hexane to be analyzed by TLC and HPLC.

#### 2.4.1. TLC Analysis

To quantify and visualize the cholesterol degrading potency of each bacterial strain, extracted samples were applied on the silica gel plates. Different dilutions of cholesterol (0.1-1.0 mg/mL) were applied on the same silica plate to compare the spot intensity. Silica plates were set in a chromatography tank containing hexane/ethyl acetate (1:1). The dried plates were sprayed with 15% phosphomolybdic acid, and spots of varying intensity were developed.

#### 2.4.2. HPLC Analysis

To verify the cholesterol degradation, extracted sample was redissolved in hexane and filtered, and 20 *μ*L was analyzed by reverse phase HPLC using Waters HPLC system with UV detector and C18 column, at* ƛ* 210 nm and hexane/acetonitrile (96:4) was used as mobile phase with a flow rate of 0.72 mL/min.

### 2.5. Qualitative Analysis of Cholesterol Oxidase

#### 2.5.1. Colony Staining Method

Colony staining method was performed on the selected strains to confirm their cholesterol oxidase production. Filter discs were immersed into a mixture containing 6% phenol, 1.7 % 4-aminoantipyrine, 0.5% cholesterol, and 3U/mL horse radish peroxidase (HRP) in 100 mM potassium phosphate buffer (pH 7.0). After that, filter discs were placed on fresh colonies of* B. pumilus *W8 and* S. marcescens *W1 and incubated at 37°C for 24 hrs. The appearance of red color is the confirmation of cholesterol oxidase production because of the formation of quinoneimine dye [27].

#### 2.5.2. Cholesterol Oxidase Indicator Plates

Cholesterol oxidase producing* B. pumilus* W8 and* S. marcescens* W1 was selected on indicator plates containing 1.0 g/L Triton X-100, 1.0 g/L cholesterol, 0.1 g/L o-dianisidine, and 1U/mL horse radish peroxidase of agar medium. On these plates, cultures were streaked and incubated at 37°C. Cholesterol oxidase from bacteria converts the cholesterol into hydrogen peroxide (H_2_O_2_), changes the medium color to intense brown due to formation of azo compound, and was taken as positive result.

### 2.6. Quantitative Analysis of Cholesterol Oxidase

#### 2.6.1. Enzyme Assay Method I

The activity of cholesterol oxidase was measured by generation of H_2_O_2_ [28]. The assay mixture (total 1 mL) consisted of 87 mM potassium phosphate buffer, 0.89 mM cholesterol, 64 mM sodium cholate, 1.4 mM 4-aminoantipyrine, 21 mM phenol, 0.34 % tween 80, and horse radish peroxidase 5 U/mL. The reaction mixture was incubated with 100 *μ*L of enzyme at 35°C for 5 minutes, and the formation of quinoneimine dye was tracked by checking the absorbance at *λ* 500 nm.

The activity of enzyme was calculated according to following formula:

(2) Unit/mL=ΔOD/minΔOD  test−ΔOD  blank13.78×Vs×Vt×df

where Vt is the total volume (1 mL) of assay, 13.78 is the millimolar extinction coefficient of quinoneimine dye, df is the dilution factor, and Vs is the enzyme volume (0.1 mL) used in assay.

#### 2.6.2. Enzyme Assay Method II

Enzyme assay was also accomplished by measuring the degradation of cholesterol into cholest-4-en-3-one. Cholesterol solution (1 mL) was mixed with 1 mL of phosphate buffer. Spectrophotometer was calibrated against the same buffer blank, then 0.1 mL of crude enzyme was added and incubated in a shaker incubator for 1 minute at 35°C, then 20 *μ*L of triton X-100 was added in order to stop the reaction, and enzyme activity was measured at* ƛ* 240 nm and calculated according to the following formula:

(3) Enzyme  activity  unit/mL=ΔOD×Total  volume×0.082Volume  of  enzyme  taken

### 2.7. Protein Estimation

Method of Lowry et al. [29] was used for estimation of total protein content. Bovine serum albumin was taken as standard.

### 2.8. Purification of Enzyme

The extracellular enzyme from the fermentation broth of the selected strains was purified by solvent precipitation using acetone. Precipitation was performed by using different broth to acetone ratios such as 1:1, 1:2, 1:3, and 1:4. In phosphate buffer the precipitated enzyme was resuspended and kept at 4°C [30].

## 3. Results

### 3.1. Screening of Isolated Microorganisms

Several bacterial strains were checked for their growth on M9 salt media. Some bacterial isolates utilized cholesterol and grown well on M9 cholesterol agar media. On the basis of maximum growth,* B. pumilus* W8 and* S. marcescens* W1 were selected for optimization studies and cholesterol oxidase production.

### 3.2. Degradation of Cholesterol by* B. pumilus *W8 and* S. marcescens* W1

**Figure 1:**
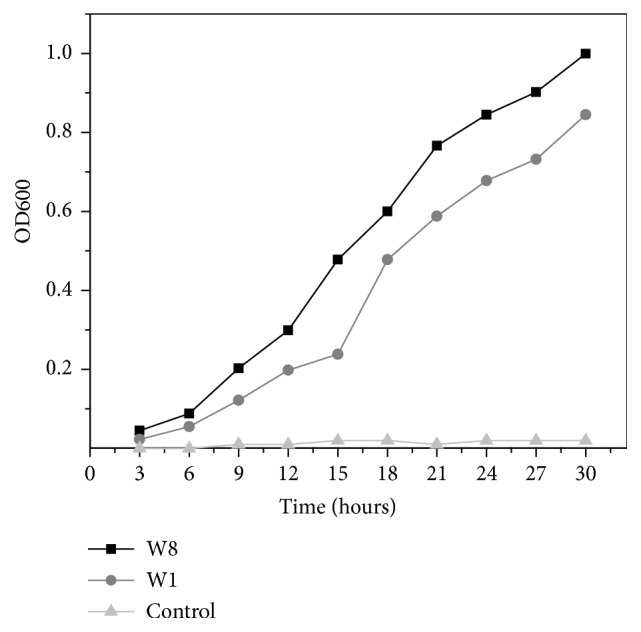
Growth of the* Bacillus pumilus* W8 and* Serratia marcescens* W1 in M9 media with cholesterol as carbon source.

The selected bacterial isolates were inoculated in M9 broth and their growth was determined by increase in OD_600_. The gradual increase in the growth was observed and the absorption at 600 nm reached 0.845 and 0.999 for* S. marcescens* W1 and* B. pumilus *W8, respectively, in 30 hours (Figure 1).

### 3.3. Optimization of Process Variables

#### 3.3.1. Incubation Period

**Figure 2:**
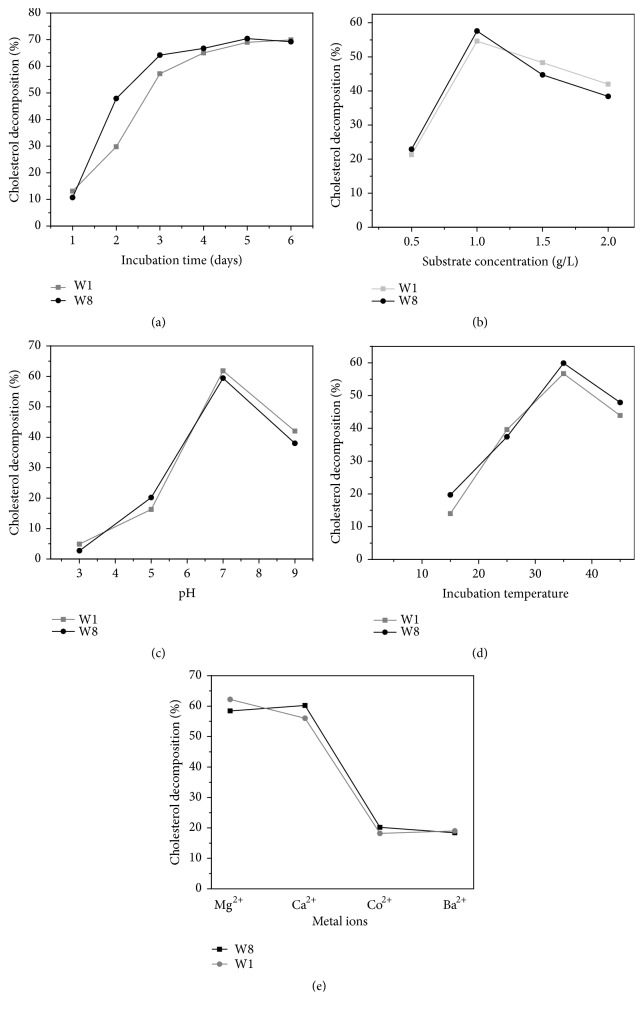
Effect of different parameters on degradation of cholesterol in M9 medium: (a) incubation time; (b) cholesterol concentration; (c) pH; (d) temperature; and (e) metal ions.

As shown in Figure 2(a), the highest degradation of cholesterol was attained on the 5^th^ day of incubation, by both the isolates. Generally, at any incubation time the decomposition of cholesterol by* B. pumilus *W8 was significantly higher as compared to* S. marcescens* W1.

#### 3.3.2. Substrate Concentration

Both the isolates* S. marcescens *W1 and* B. pumilus *W8 showed increased cholesterol degradation with increasing concentration of cholesterol from 0.5 up to 1.0 g/L, while further increase in concentration of cholesterol reduced the degradation (Figure 2(b)). Maximum degradation of cholesterol (54.6% and 57.6%) was observed with* Serratia marcescens* W1 and* Bacillus pumilus* W8 at a concentration of 1.0 g/L.

#### 3.3.3. pH

The most favorable pH for decomposition of cholesterol by* S. marcescens* W1 and* B. pumilus* W8 was pH 7.0 (Figure 2(c)). The activities of both the isolates decreased towards alkaline and acidic sides. The cholesterol decomposing activity was better at moderate alkaline pH (9.0) in comparison with acidic (5.0). At the extremely acidic pH (pH 3.0) both* S. marcescens* W1 and* B. pumilus* W8 did not grow well.

#### 3.3.4. Incubation Temperature

With increasing temperature from 15 to 35°C, the cholesterol decomposition significantly increased, and with further increase in temperature, the cholesterol decomposing activity decreased (Figure 2(d)).

#### 3.3.5. Metal Ions

Mg^+2^ and Ca^+2^ were the most appropriate metallic ions for cholesterol decomposing activity. The maximum reduction was recorded with MgSO_4_ and CaCl_2_ by both* Bacillus pumilus *W8 and* Serratia marcescens* W1. BaCl_2_ and CoCl_2_ also showed positive effect on cholesterol degradation but less as compared to Mg^+2^ and Ca^+2^ for both the tested isolates (Figure 2(e)).

**Figure 3:**
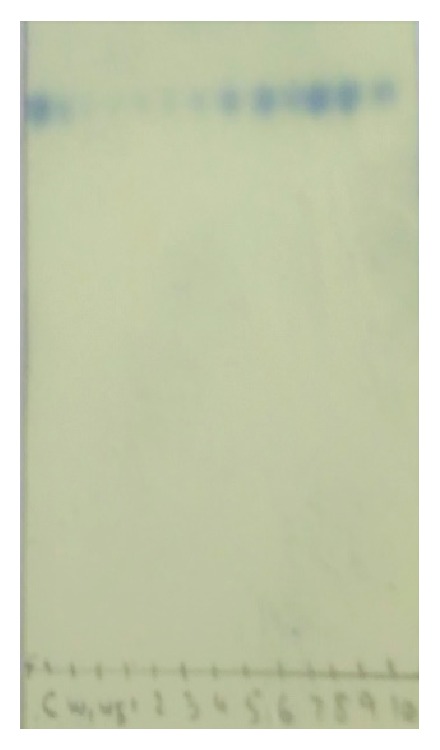
TLC of cholesterol degradation by* Serratia marcescens *W1 and* Bacillus pumilus* W8 and its comparison with control (C) and with different concentration of cholesterol (0.1-1.0 g/L).

TLC analysis was carried out in order to determine whether cholesterol level decreased and number of metabolites produced during the growth of bacterial strains. The transformation extracts showed single spot with low intensity, indicating the utilization of cholesterol with no accumulation of metabolites (Figure 3).

**Figure 4:**
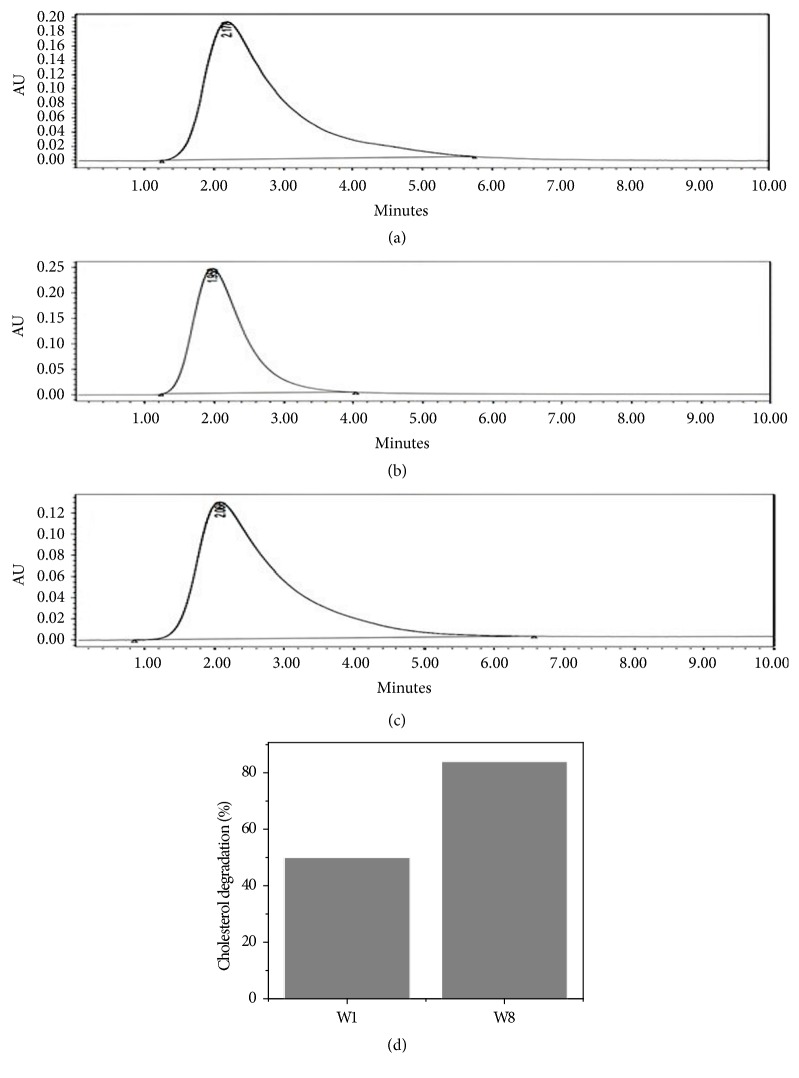
HPLC analysis of cholesterol degradation: (a) zero (0) time; (b)* Serratia marcescens* W1 sample after 4 days; (c)* Bacillus pumilus* W8 sample after days; (d) % degradation.

Degradation of cholesterol was observed with* Serratia marcescens* W1 and* Bacillus pumilus* W8 on the basis of HPLC analysis (Figures 4(a), 4(b), and 4(c)). The strains W1 and W8 cleared 50% and 84% of cholesterol, respectively, after four days of incubation (Figure 4(d)).

**Figure 5:**
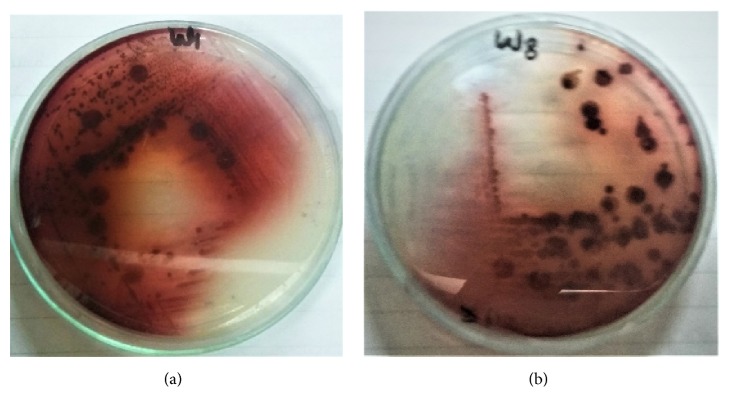
Development of red color in colony staining method due to cholesterol oxidase production: (a)* Serratia marcescens* W1, (b)* Bacillus pumilus* W8.

**Figure 6:**
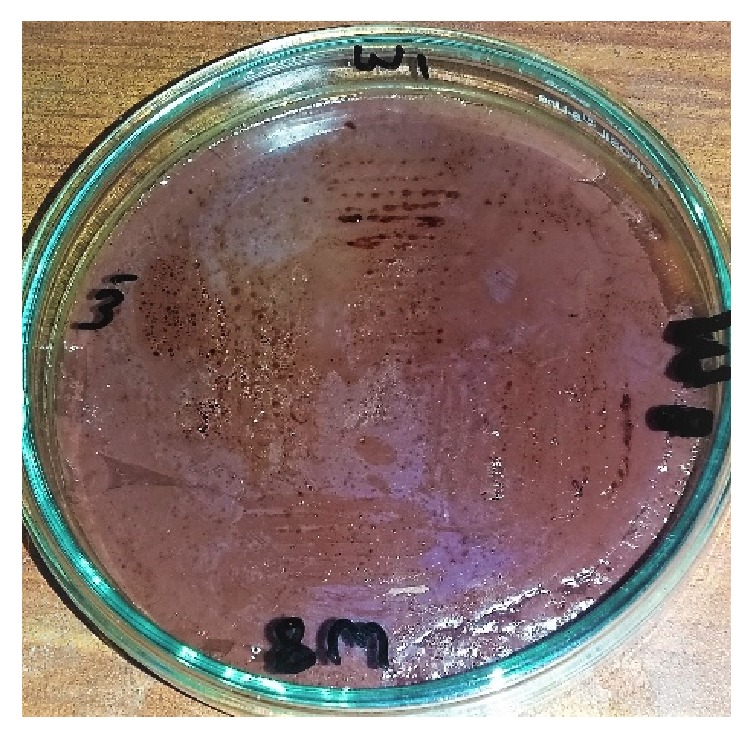
Growth of* Serratia marcescens* W1 and* Bacillus pumilus* W8 on cholesterol oxidase indicator plate.

Cholesterol oxidase production was assessed by quantitative and qualitative method. For qualitative analysis, cholesterol oxidase indicator plate sssay and colony staining method were carried out. In colony staining cholesterol oxidase was confirmed by formation of red color (Figures 5 and 6).

While in the indicator plate assay the development of azo component turns the medium into intense brown due to the presence of H_2_O_2_ produced by reaction of cholesterol oxidase (Figure 6).

For quantitative analysis cholesterol oxidase was purified by solvent extraction at acetone to broth (1:1) ratio. The activity of cholesterol oxidase isolated from culture supernatant of* B. pumilus *W8 and* S. marcescens* W1 was recorded to be 1.64 U/mL and 1.47 U/mL as compared to crude which was 1.26 U/mL and 1.03 U/mL, respectively.

## 4. Discussion

Intracellular accumulations of recalcitrant substances impair the cell viability and function, leading to development of macular degeneration, atherosclerosis, and neurodegenerative diseases [31]. Accumulation of cholesterol is linked to some of the diseases including stroke, coronary heart disease, peripheral vascular disease, high blood pressure, and diabetes. Elevated serum cholesterol is usually regarded as a risk factor for other diseases depending on the type of blood vessels which are blocked or narrowed [32].

Oxidized sterols preferentially generated by the oxidation of cholesterol. 7-Ketocholesterol is the oxidized derivative of cholesterol formed by the free radical attack on carbon 7 of the cholesterol. Elevated concentrations of the oxysterol are linked with decreased cell viability, disruption of cellular homeostasis, and increased cell death [33].

From diverse habitat, several bacteria and their enzymes have been reported to have the ability to degrade cholesterol and 7-ketocholesterol. The degradation of these compounds is initiated by most of these species with the same reaction mechanism as cholesterol oxidase [34].

In the present study bacteria were isolated from oil-contaminated sites and showed the activity for cholesterol degradation and cholesterol oxidase production. Two of the isolates,* B. pumilus* W8 and* S. marcescens *W1, were showing maximum activity for cholesterol degradation. Saranya et al. [35]. isolated the* Bacillus *sp. from the oil and soap industrial wastes, with a potential to degrade the cholesterol. Several species of* Bacillus* and* Serratia* are reported to have cholesterol degrading ability [27, 36].

Regarding the effect of incubation time, the maximum cholesterol degrading activity was detected after four days of incubation. The incubation time varies in different microorganisms for cholesterol decomposition. The maximum cholesterol utilization by* Bacillus cereus* was attained after 24 hrs incubation [27], while the others reported 3-7 days for the maximum degradation of cholesterol by most of* Rhodococcus *strains [37].

Some microorganisms effectively degraded the cholesterol based on the temperature. The strains in the present study, W1 and W8, degraded maximum cholesterol at 35°C. During the optimization studies of* Bacillus subtilis* strain, KAVK3 degraded maximum cholesterol at 35°C [38].

The maximum cholesterol degradation by both the tested isolates was attained at pH 7.0. The cholesterol decomposing activity was steadily decreased with pH shift away from the optimum. Similarly, it has been reported previously that pH 7.0 and 6.5 are the optimum pH for cholesterol degradation and cholesterol oxidase production by* Bacillus sp. *[35, 38].

In the current work cholesterol was used as the only carbon source, and its varying concentrations influence the degradation. The maximum degradation was achieved at concentration of 1.0 g/L. However, on increasing the cholesterol concentration, the degrading activity by the selected isolates significantly decreased. Ouf et al. [39] reported similar results; that is, the low concentrations were degraded more rapidly than higher concentrations of cholesterol by* Streptomyces. *Similarly optimum cholesterol concentration (1mg/mL) for degradation by* Bacillus cereus *was reported by Kuppusamay and Kumar [27].

For cholesterol degradation the most suitable metallic ions for* B. pumilus *W8 and* S. marcescens *W1 were MgSO_4_ and CaCl_2_, whereas BaCl_2_ and CoCl_2_ induced low activity for both the tested isolates. These findings are in agreement with Ouf et al. [39] who found that the most suitable ions for cholesterol degradation were MgSO_4_ followed by CaCl_2_ for treated and untreated* S. fradiae *bacteria, while for the enzymes recovered from* Bacillus cereus* and* Pseudonocardia compacta*, the most stimulatory ion was MgSO_4_ followed by ZnSO_4_ [27, 40].

Based on HPLC result, 50 % and 84 % cholesterol reduction were achieved by* S. marcescens* W1 and* B*.* pumilus* W8, respectively. There was also no other peak present after the degradation in the tested samples indicating their complete degradation (Figures 4(b) and 4(c)). The degrading activity of the tested isolates was mostly because of production of extracellular cholesterol oxidase (CHO). In catabolic pathway of cholesterol, many microorganisms produce the cholesterol oxidase as an initial enzyme. The isolates tested in the present study also showed cholesterol oxidase activities in both qualitative and quantitative assays.

We have also tested* Bacillus pumilus* W8 and* Serratia marcescens* W1 for degradation of 7-ketocholesterol, and significant reduction was achieved (unpublished data).

## 5. Conclusion

The present study showed that the cholesterol degrading microorganisms from the oil-contaminated soil are endowed with the capability to degrade the cholesterol and are good source for cholesterol oxidase that can be exploited for potential biomedical, industrial, environmental, and industrial applications. Overall, these findings suggest that the biodegradation processes may control the levels of cholesterol. As cholesterol and its oxidized derivatives are associated with a number of aging diseases, its removal by exogenous catabolic enzymes can reverse the disease conditions.

## Data Availability

The data used to support the findings of this study are available from the corresponding author upon request.

## Supplementary Materials

Supplementary Materials Graphical abstract describing the degradation of cholesterol by bacterial isolates.
