# Supplementary material for: Cholesterol Degradation and Production of Extracellular Cholesterol Oxidase from Bacillus pumilus W1 and Serratia marcescens W8
Source: Biomed Res Int. 2019 Apr 28;2019:1359528. doi: 10.1155/2019/1359528 (PMC6512041; doi:10.1155/2019/1359528)
Supplement: Supplementary Materials — Graphical abstract describing the degradation of cholesterol by bacterial isolates. [file 1359528.f1.pdf]

***Cholesterol degradation and production of extracellular cholesterol oxidase from Bacillus pumilus* W1 *and Serratia marcescens* W8.**

Graphical Abstract

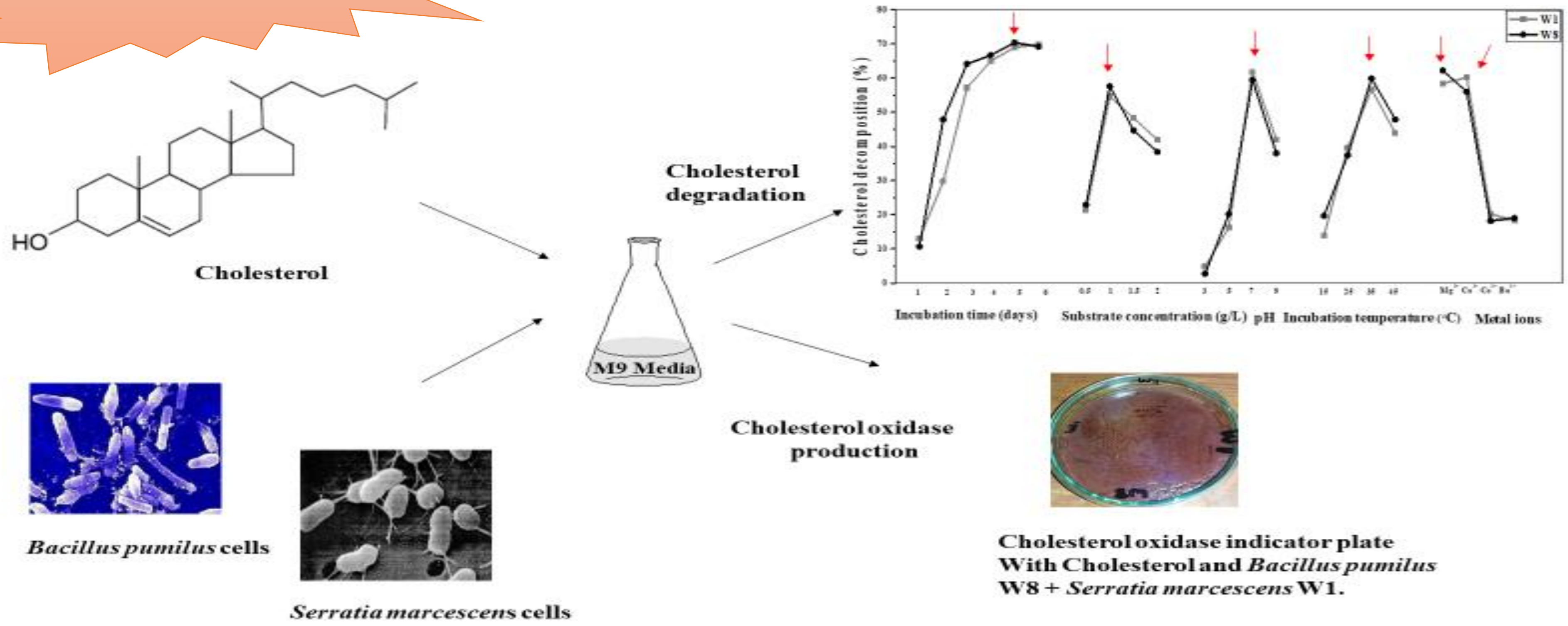

The paper describes degradation of cholesterol by bacterial isolates. The enzyme in the process was identified as cholesterol oxidase by plate assay producing color on indicator plates. The degradation of cholesterol was the maximum at 5 days of incubation, with 1g/L substrate concentration, at pH 7.0 and 35˚C in M9 medium with cholesterol as sole carbon and energy source. Metal ions, Ca^{+2} and Mg^{+2} had positive effect while Co^{+2} and Zn^{+2} showed negative effect on degradation. The study revealed potential of bacterial enzymes for medical bioremediation of age related products in the body.
